# Polymyositis Presenting With Respiratory Failure and Cardiac Arrest: A Case Report

**DOI:** 10.7759/cureus.82887

**Published:** 2025-04-24

**Authors:** Jeehad M Felemban, Ammar Z Faloudah, Murad M Mawlawi, Ahmed Y Shebly, Mohannad AbuRageila

**Affiliations:** 1 Adult Critical Care Medicine, Security Forces Hospital, Makkah, SAU; 2 Adult Critical Care Medicine, King Abdullah Medical City, Makkah, SAU; 3 Pulmonary and Adult Critical Care Medicine, Security Forces Hospital, Makkah, SAU

**Keywords:** acute polymyositis, adult cardiac arrest, autoimmune myopathy, critical care, multidisciplinary care approach, severe respiratory failure

## Abstract

This case report presents a 30-year-old woman who experienced respiratory failure and cardiac arrest as a complication of polymyositis, which is an idiopathic inflammatory myopathy disease. Autoimmune diseases, such as polymyositis, can cause respiratory failure due to respiratory muscle weakness or interstitial lung involvement, leading to significant complications if not promptly addressed. The patient's complex clinical presentation included progressive muscle weakness, respiratory distress, and cardiac instability, underscoring the necessity for a comprehensive diagnostic approach. The report details the patient's clinical course, diagnostic workup, and multidisciplinary management, which involved immunosuppressive therapy, mechanical ventilation, and physiotherapy. This case emphasizes the critical role of a coordinated, multidisciplinary team in diagnosing and managing severe autoimmune diseases with multisystem involvement.

## Introduction

Polymyositis is an idiopathic inflammatory myopathy characterized by the subacute onset of proximal, symmetrical muscle weakness, elevated muscle enzymes, and muscle inflammation evident on biopsy. It belongs to a broader group of diseases known as idiopathic inflammatory myopathies [1]. Notably, dermatomyositis, a subtype of idiopathic inflammatory myopathies distinguished by characteristic skin findings (e.g., heliotrope rash and Gottron's papules), differs from polymyositis, which lacks these specific cutaneous manifestations and is diagnosed primarily through muscle biopsy demonstrating endomysial inflammation; selective proximal muscle weakness and the absence of inclusion bodies further differentiate it from other idiopathic inflammatory myopathies [2]. Polymyositis affects about 1-8 cases per 100,000 individuals [3]. Although progressive muscle weakness is the hallmark of polymyositis, the disease can also present with extra-muscular manifestations with severe complications affecting the cardiac and respiratory systems, primarily due to diaphragmatic paralysis and muscle weakness [4-6]. While clinically significant cardiac involvement secondary to respiratory failure or autonomic dysregulation is rare, subclinical manifestations such as myocarditis, pericarditis, cardiomyopathy, conduction abnormalities, and arrhythmias are more frequently observed. Respiratory complications are common and often include interstitial lung disease and aspiration pneumonia, resulting from pharyngeal muscle involvement [4-8]. However, respiratory failure or significant dyspnea due to ventilatory muscle weakness remains rare, affecting fewer than 5% of patients [7]. This case report is particularly unique as it involves both respiratory failure and cardiac arrest, a combination not commonly seen in polymyositis. The progression to sudden cardiac arrest underscores the severe systemic impact of the disease, particularly with diaphragmatic involvement and autonomic dysregulation, making this case exceptional in highlighting the importance of recognizing and managing the diverse and severe manifestations of the disease.

## Case presentation

A 30-year-old woman with a known benign neck mass and a recent history of thumb surgery presented with a three-month history of progressive, generalized muscle weakness, accompanied by dyspnea and dysphagia. She also developed progressive facial puffiness, periorbital edema, and swelling of the limbs, along with chest and shoulder rashes which are characterized by multiple small, discrete, erythematous macules. These symptoms progressively worsened, ultimately resulting in severe orthopnea, requiring her to sleep in a semi-sitting position. Upon presentation at the emergency department, the patient was in respiratory distress, with the following vital signs: heart rate, 128 bpm; blood pressure, 105/70 mmHg; respiratory rate, 28 breaths per minute; oxygen saturation, 89% in room air, improving to 95% with supplemental oxygen; and temperature, 37.1°C. Neurological examination revealed significant proximal muscle weakness, graded 3/5 in both upper and lower limbs on the Medical Research Council Scale. Distal muscle strength remained intact (5/5). Cranial nerve examination showed mild dysphagia without facial weakness. Deep tendon reflexes were hyporeflexic in the lower limbs but preserved in the upper limbs. No sensory deficits were noted. A cardiac examination demonstrated tachycardia with a regular rhythm. Heart sounds were normal, and there was no evidence of murmurs, pericardial rubs, or signs of heart failure. Autonomic dysfunction was suggested by supine tachycardia with postural hypotension. Lung auscultation revealed clear breath sounds without crepitations or wheezing. Despite an initially unremarkable chest X-ray, the patient's worsening respiratory distress, progressive orthopnea, and tachycardia with a positive D-dimer test initially raised the suspicion of pulmonary embolism (PE) in the emergency room. During a computed tomography (CT) pulmonary angiography, the patient initially had difficulty lying flat but was able to maintain the position for imaging. However, she suddenly became unresponsive during the procedure and experienced a cardiac arrest. She was successfully resuscitated after four cycles of cardiopulmonary resuscitation, intubated, and transferred to the intensive care unit (ICU) connected to a mechanical ventilator. An echocardiogram revealed normal cardiac function, and PE was ruled out by the CT pulmonary angiography. Pre-arrest electrocardiogram (ECG) demonstrated sinus tachycardia without ischemic changes, conduction abnormalities, arrhythmias, myocarditis, or pericarditis. Post-resuscitation, the ECG continued to exhibit sinus tachycardia without new alterations, thereby excluding a persistent arrhythmic etiology. A multidisciplinary meeting involving rheumatology and neurology was held to guide further diagnostic and management strategies and to evaluate the patient's constellation of symptoms, which included progressive muscle weakness, dyspnea, dysphagia, facial and limb swelling, and chest rashes.

Diagnostic workup

**Figure 1:**
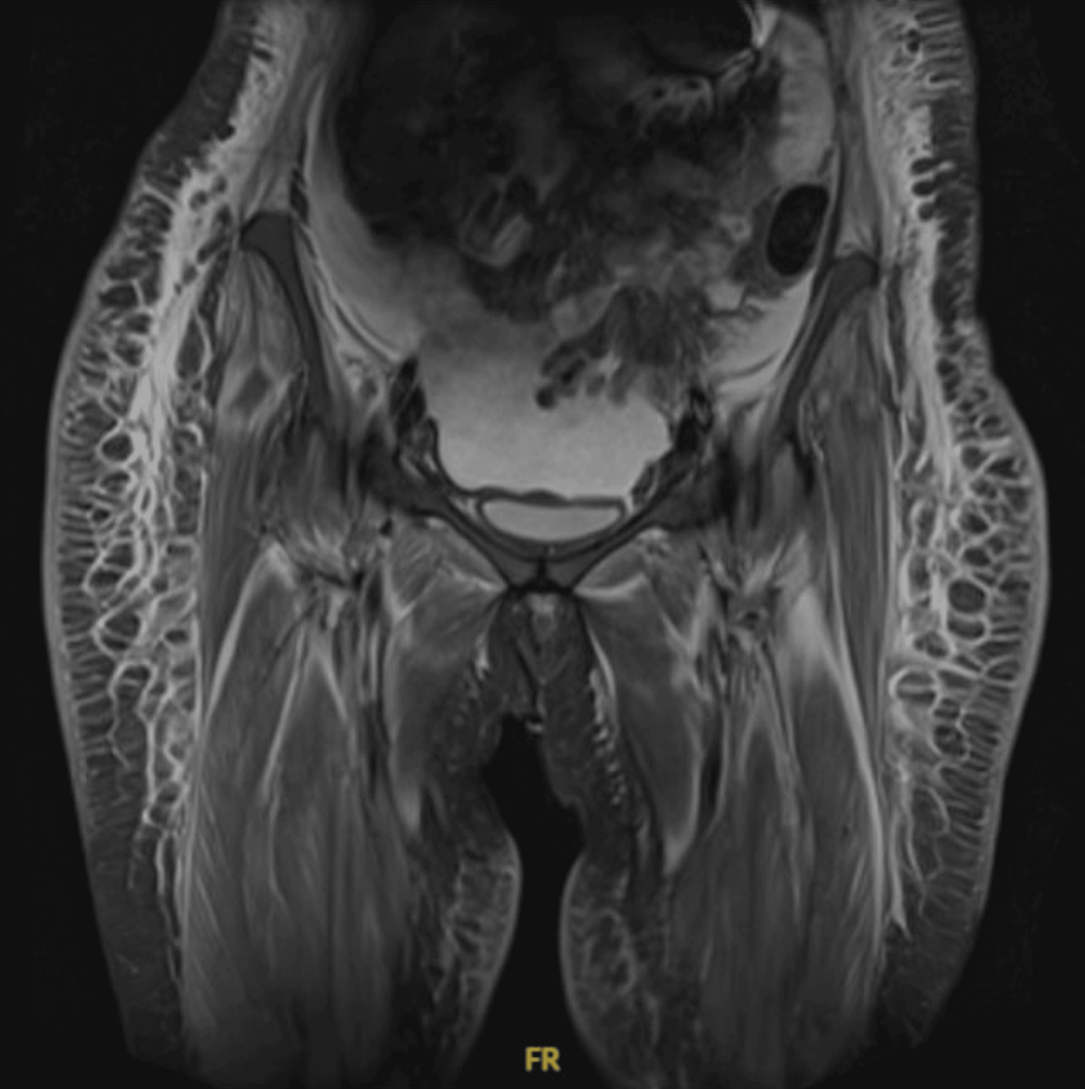
MRI of the bilateral thighs Bilateral thighs with extensive subcutaneous and muscle edema affecting the vastus lateralis and quadriceps groups MRI: magnetic resonance imaging

**Figure 2:**
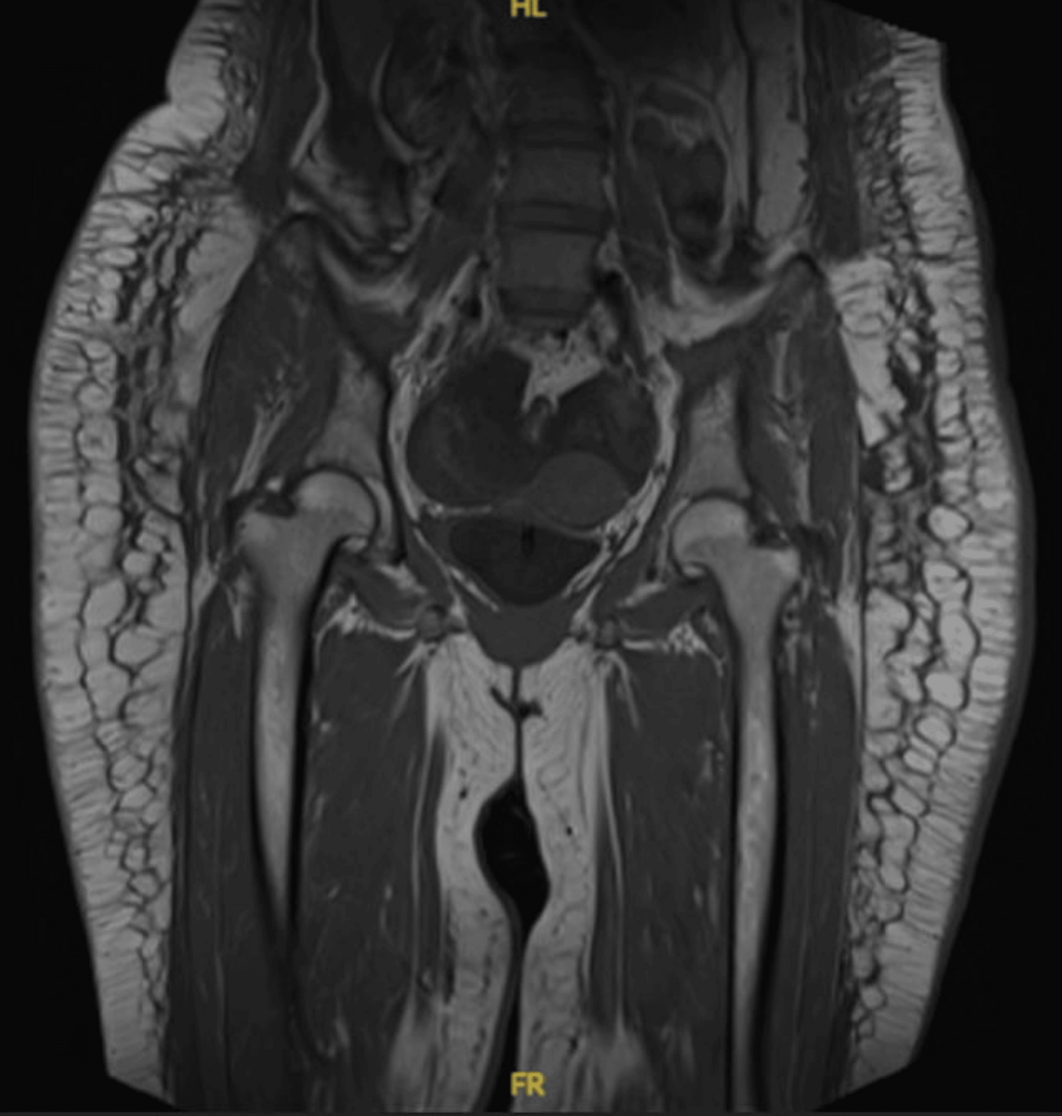
MRI of the bilateral thighs Bilateral extensive subcutaneous and muscle edema particularly affecting the proximal muscles MRI: magnetic resonance imaging

Initial laboratory investigations revealed elevated creatine kinase (CK) and lactate dehydrogenase (LDH) levels, both of which are indicative of myositis. Further autoimmune testing demonstrated a positive result for antinuclear antibody (ANA) and a strongly positive anti-Sjögren's syndrome antigen A (anti-SSA/Ro) antibody. Additionally, the rheumatoid factor (RF) was weakly positive. However, tests for several other autoimmune markers, including anti-cyclic citrullinated peptide (ACCP), lupus anticoagulant, anti-double-stranded DNA (anti-dsDNA), anti-Jo-1 antibody, anti-centromere protein (ACP), anti-Smith (anti-Sm), anti-La, autoimmune thyroid antibodies, anti-ribonucleoprotein (anti-RNP), anti-scleroderma antigen-70 (anti-SCL-70), extractable nuclear antigen (ENA), and anti-Sjögren's syndrome antigen B (anti-SSB/La) antibodies, were all negative. Complement levels C3 and C4 were found to be within normal limits. Electromyography (EMG) and nerve conduction studies (NCS) showed findings consistent with polyaxonal neuropathy/myopathy. Furthermore, a magnetic resonance imaging (MRI) of the bilateral thighs revealed extensive subcutaneous and muscle edema notably affecting the vastus lateralis and quadriceps groups, which was in keeping with myopathy (Figure 1 and Figure 2). Finally, muscle biopsy results confirmed the diagnosis of polymyositis, providing further support for the clinical suspicion.

Management and complications

The patient was initially treated with steroids at a dosage of 1 mg/kg from day 1 to day 5. This resulted in slight improvement; however, persistent respiratory muscle weakness prevented successful weaning from mechanical ventilation. She was unable to tolerate a reduction in support, specifically pressure support ventilation (PSV), and remained ventilator-dependent. To address this, pulse steroids were administered from day 5 to day 9, followed by maintenance therapy with methylprednisolone based on its rapid anti-inflammatory effects in acute settings, which was later switched to prednisolone at a dose of 50 mg daily to allow for a more gradual taper and the long‐term management of inflammation. In addition to steroids, an initial five-day course of intravenous immunoglobulin (IVIG) was given from day 1 to day 5, with additional doses on days 23, 24, and 28, totaling eight doses. Methotrexate therapy was initiated on day 10 at a dose of 15 mg weekly. However, after three doses, treatment was postponed due to the development of nosocomial infections and the concerns regarding its broader immunosuppressive effects such as the potential bone marrow suppression during active infection. Rituximab therapy began on day 16 after the patient screened negative for latent tuberculosis and hepatitis. Despite these interventions, the patient experienced ongoing respiratory muscle weakness, which led to failed extubation attempts on two occasions. A tracheostomy was recommended but declined by both the patient and her family. A gradual weaning strategy was implemented, including both PSV and volume support ventilation (VSV). During this period, the patient showed gradual improvement, as evidenced by an increasing negative inspiratory force (−20 cm H₂O). Successful extubation occurred on day 28, following the achievement of appropriate respiratory parameters. Post-extubation, the patient was transitioned to non-invasive ventilation (NIV), alternating with a high-flow nasal cannula (HFNC), and later weaned to room air without complications. Early initiation of physiotherapy, including respiratory muscle training and a gradual mobilization plan, was implemented as part of the patient’s comprehensive rehabilitation strategy. Sessions were conducted daily, targeting airway clearance, breathing exercises, and ambulation which led to significant improvements in both proximal and distal muscle strength, facilitating ambulation and further enhancing respiratory function. During her hospital stay, the patient developed several nosocomial infections. She was diagnosed with aspiration pneumonia, which was treated with a seven-day course of antibiotics. Initial sputum cultures revealed the presence of *Streptococcus pneumoniae*, while follow-up cultures on days 22 and 32 identified colonization with *Pseudomonas aeruginosa* and *Stenotrophomonas maltophilia*, both of which were managed conservatively as the patient remained asymptomatic. Additionally, a urinary tract infection caused by AmpC-positive *Enterobacter cloacae* was successfully treated with a 10-day course of antibiotics, with follow-up cultures returning negative. Through a multidisciplinary approach including immunosuppressive therapy, tailored mechanical ventilation strategies, physiotherapy, and vigilant infection control, the patient showed significant clinical improvement. She was eventually discharged with plans for ongoing physiotherapy and outpatient follow-up.

Outcome

The patient showed significant improvement in both muscle strength and respiratory function, which ultimately enabled successful extubation and ambulation. Upon discharge, she was prescribed ongoing physiotherapy and scheduled for regular outpatient follow-up about three months after ICU discharge. During the subsequent clinic visits, she was found to be in remission with a complete resolution of muscle weakness and dyspnea, normalization of CK and inflammatory markers, and return to independent ambulation. At that time, she continued to receive IVIG and rituximab to prevent relapse. One and a half years after discharge, on March 31, 2024, the patient remained in remission and was admitted for a scheduled course of IVIG. She received a five-day course of IVIG as part of a maintenance protocol triggered by a mild CK rise, despite the absence of clinical relapse, and was discharged thereafter, continuing her treatment regimen.

## Discussion

Polymyositis is a rare but serious cause of respiratory failure and cardiac arrest. Its diagnosis can be particularly challenging due to the overlap of symptoms with other autoimmune and neuromuscular diseases [1]. This case highlights the importance of a comprehensive diagnostic approach and a multidisciplinary management strategy to optimize patient outcomes. Early initiation of immunosuppressive therapy and supportive care were critical in improving this patient's condition, as demonstrated in several case reports and studies [4,7,8]. This patient presented a significant diagnostic challenge because of the muscle weakness and respiratory involvement, which are characteristics of polymyositis but can also overlap with other conditions like inclusion body myositis, dermatomyositis, and even neuromuscular diseases such as Sjögren's syndrome [1]. The initial differential diagnosis was complicated by these overlapping features. For example, dermatomyositis typically presents with cutaneous symptoms, but in some cases, especially those with minimal skin involvement, the disease may resemble polymyositis, as seen in a study by Syue et al. [9], who reported a similar diagnostic dilemma. This underscores the importance of thorough clinical evaluation and autoimmune workup, including serological tests for ANA and anti-SSA/Ro antibodies, as performed in this case. MRI played a pivotal role in this patient's diagnosis. In this case, MRI findings correlated well with the clinical presentation and laboratory results, reinforcing the diagnosis of polymyositis. MRI of the thighs revealed extensive subcutaneous and muscle edema, which is a hallmark of polymyositis [5]. Unlike other imaging modalities, MRI provides superior soft-tissue resolution, enabling the early detection of muscle inflammation and edema even before clinical symptoms fully develop. Similar findings were reported in a study by Dauriat et al. [5], where MRI was instrumental in diagnosing diaphragmatic weakness in a patient with polymyositis. Moreover, muscle biopsy played a crucial role in confirming the diagnosis of polymyositis in this patient. Muscle biopsy confirmed the diagnosis by revealing characteristic histopathological features such as endomysial inflammation, muscle fiber necrosis, and muscle fiber regeneration, which are consistent with polymyositis. This is in line with findings from Kalluri and Oddis [7], who emphasized the importance of muscle biopsy in cases where clinical presentation overlaps with other inflammatory myopathies. Utilizing the European League Against Rheumatism/American College of Rheumatology (EULAR/ACR) classification criteria for adult and juvenile idiopathic inflammatory myopathies and their major subgroups, which include a combination of clinical, laboratory, and histopathological features, we were able to systematically assess and diagnose this patient accurately. The biopsy findings, along with elevated muscle enzymes and positive autoimmune markers (ANA, anti-SSA/Ro antibody), met the classification criteria for polymyositis, thereby solidifying the diagnosis [2]. This comprehensive approach highlights the importance of muscle biopsy in the diagnostic algorithm of idiopathic inflammatory myopathies, ensuring the precise identification and appropriate management of the disease. Respiratory failure due to respiratory muscle weakness is a major complication of polymyositis. Similar to findings reported by Selva-O'Callaghan et al. [4], this patient initially required mechanical ventilation due to diaphragmatic paralysis. Mechanical ventilation is often required in patients with significant respiratory muscle involvement, as it provides support until muscle strength improves. Our patient was successfully weaned from mechanical ventilation, transitioning from bilevel positive airway pressure (BiPAP) to continuous positive airway pressure (CPAP) and eventually to room air, demonstrating significant recovery in respiratory function. Dauriat et al. [5] noted that diaphragmatic weakness can be a key feature of respiratory involvement in polymyositis and early detection is crucial for managing respiratory failure. The gradual weaning process in our case, aided by physiotherapy and muscle strength improvement, aligns with recommendations from Kalluri and Oddis [7], who reported similar successful extubations after prolonged ventilator support in polymyositis patients. Cardiac arrest in polymyositis is a rare but serious complication, often linked to diaphragmatic weakness and its impact on autonomic nervous system regulation [6,8]. The cardiac arrest in this patient can be attributed to the unique interplay of diaphragmatic paralysis and its impact on cardiac conduction and rhythm. The presence of these disturbances predominantly in the supine position suggests a mechanical influence on the cardiac plexuses. The bilateral diaphragmatic paralysis likely caused the compression of the vegetative cardiac plexuses (a network of autonomic nerves at the base of the heart, comprising sympathetic and parasympathetic fibers that regulate heart rate, rhythm, and conduction) and upward traction of the celiac plexus due to the elevated diaphragm, leading to parasympathetic stimulation. This mechanism is consistent with studies by Dregoesc et al. [8] and Srivatsav et al. [6], who reported similar occurrences of cardiac arrhythmias and cardiac arrest in polymyositis patients with diaphragmatic involvement. Additionally, severe hypoxemia exacerbated by diaphragmatic weakness may have further precipitated cardiac arrest, a scenario observed by Ramana Raju et al. [10] in a case of seronegative polymyositis. In the present case, the patient may have been able to maintain acceptable breathing while upright due to the effect of gravity assisting diaphragmatic function. However, when lying flat, the loss of gravitational assistance likely exacerbated respiratory compromise, leading to significant hypoxemia and subsequent cardiac arrest. Cardiac involvement in polymyositis is an area that is still under-explored, but several studies have highlighted its significance. Ramana Raju et al. [10] and recent reviews on cardiac manifestations of inflammatory myopathies suggest that patients with polymyositis may present with a range of cardiac arrhythmias and even heart failure [11]. This supports the need for careful cardiac monitoring in polymyositis patients, particularly those with significant diaphragmatic dysfunction. The management of polymyositis can be complicated by concurrent infections, as was evident in this case. While high-dose corticosteroids and immunosuppressive agents like methotrexate and IVIG are essential to manage the underlying autoimmune process, these treatments can predispose patients to infections [4,7]. In this case, methotrexate therapy was delayed due to nosocomial infections, highlighting the challenge of balancing immunosuppression with the need for infection control. This is consistent with the findings of Syue et al. [9], who highlighted the need for careful monitoring and adjustment of immunosuppressive therapy in polymyositis patients with infections. The use of IVIG and rituximab, in this case, helped manage disease activity while minimizing the risk of infection, which aligns with recent studies advocating for the early use of biological agents in polymyositis patients with severe disease [7,8]. The multidisciplinary approach was fundamental in the effective management and diagnosis of this complex case of polymyositis. Collaboration between various specialties, including neurology and rheumatology, enabled a comprehensive and cohesive treatment strategy. The neurology team was pivotal in assessing and managing the neuromuscular complications, conducting NCS and EMG, which provided critical insights into the extent of muscle involvement. Concurrently, the rheumatology team played a crucial role in the autoimmune workup, interpreting serological tests and guiding immunosuppressive therapy. Regular multidisciplinary meetings facilitated coordinated care, allowing for timely adjustments in the treatment plan, such as the transition from initial steroid therapy to more aggressive immunosuppressive treatments like IVIG and rituximab. This integrated approach not only optimized patient outcomes but also ensured that all potential complications, such as respiratory failure and cardiac involvement, were promptly addressed. The early involvement of physiotherapists further contributed to the patient's recovery, highlighting the value of holistic and collaborative medical practice in managing rare and severe autoimmune conditions, as also emphasized in other case reports [9,11,12]. Future studies should investigate the pathophysiological basis of cardiac involvement in polymyositis, specifically examining the contributions of autonomic dysregulation, diaphragmatic compromise, and direct myocardial inflammation, to inform targeted monitoring strategies and optimize therapeutic interventions.

## Conclusions

This case illustrates the complexity and severity of polymyositis with systemic involvement, particularly when significant diaphragmatic weakness contributes to both respiratory failure and cardiac arrest. A comprehensive diagnostic workup, including elevated CK levels, MRI findings demonstrating extensive muscle edema, and confirmatory muscle biopsy, was pivotal in establishing the diagnosis and guiding management. Moreover, the use of IVIG and rituximab was instrumental in achieving rapid clinical improvement and maintaining remission, underscoring their value in the therapeutic regimen. We also encountered several challenges, such as difficulties in weaning the patient from mechanical ventilation due to persistent respiratory muscle weakness and managing nosocomial infections while on immunosuppressive therapy. Highlighting these obstacles, along with our approach to overcoming them, may provide valuable insights for other physicians managing similar cases. Overall, our findings emphasize the importance of a multidisciplinary approach in managing complex cases of polymyositis and suggest that early, coordinated diagnostic and therapeutic interventions can significantly influence patient outcomes. Further research is warranted to deepen our understanding of the pathophysiological mechanisms underlying severe polymyositis and to refine targeted treatment strategies for this challenging condition.
